# The Multifunctional Effects of Nobiletin and Its Metabolites* In Vivo* and* In Vitro*

**DOI:** 10.1155/2016/2918796

**Published:** 2016-09-27

**Authors:** Hao Huang, Linfu Li, Weimei Shi, Hai Liu, Jianqiong Yang, Xiaoliang Yuan, Longhuo Wu

**Affiliations:** ^1^College of Pharmacy, Gannan Medical University, Ganzhou 341000, China; ^2^Department of Clinical Research Center, The First Affiliated Hospital of Gannan Medical University, Ganzhou 341000, China; ^3^Department of Respiratory Medicine, The First Affiliated Hospital of Gannan Medical University, Ganzhou 341000, China

## Abstract

Nobiletin (NOB) chemically known as 5,6,7,8,3′,4′-hexamethoxyflavone is a dietary polymethoxylated flavonoid found in* Citrus* fruits. Recent evidences show that NOB is a multifunctional pharmaceutical agent. The various pharmacological activities of NOB include neuroprotection, cardiovascular protection, antimetabolic disorder, anticancer, anti-inflammation, and antioxidation. These events may be underpinned by modulation of signaling cascades, including PKA/ERK/MEK/CREB, NF-*κ*B, MAPK, Ca^2+^/CaMKII, PI3K/Akt1/2, HIF-1*α*, and TGF*β* signaling pathways. The metabolites may exhibit stronger beneficial effects than NOB on diseases pathogenesis. The biological activities of NOB have been clarified on many systems. This review aims to discuss the pharmacological effects of NOB with specific mechanisms of actions. NOB may become a promising candidate for potential drug development. However, further investigations of NOB on specific intracellular targets and clinical trials are still needed, especially for* in vivo* medical applications.

## 1. Introduction

**Figure 1:**
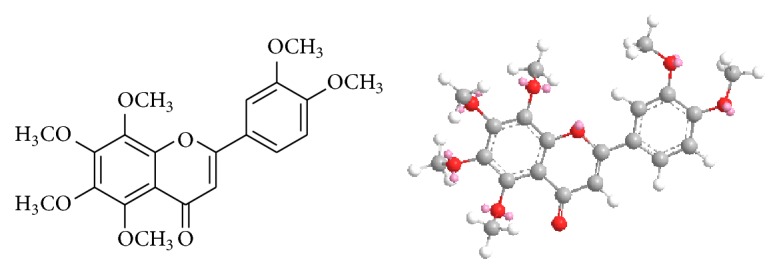
The structure of NOB (2D and 3D).

Many natural occurring compounds have become the candidates for drug development and subsequent clinic management. Nobiletin (5,6,7,8,3′,4′-hexamethoxyflavone, NOB) (Figure 1), an empirical formula of C_21_H_22_O_8_ and molecular weight of 402.39, is one of the nontoxic dietary polymethoxyflavones (PMFs) in the unique class of flavonoids mainly isolated from* Citrus* fruits [1]. The crystal structure study on NOB shows that the chromene and the arene rings are almost in the same plane. The C atoms of the two methoxy groups in the arene ring are also in the same plane, while the C atoms of four methoxy groups linking to the chromene ring are not in parallel. This conformational characteristic of chiral structure is indicated by the covalent bond rotation between the arene and the chromene rings and the conformational alternations of methoxy groups [2].

The bioactivities of a compound depend on its structure and its metabolism. Without a glycoside moiety, NOB is easily absorbed, due to its high lipophilic nature and high permeability [3], which indicates a specific mechanism. Study shows that it might be associated with an energy independently carrier-mediated (facilitated diffusion) system, which is also competitively validated for kaempferol, luteolin, quercetin, and tangeretin [4]. However, the details are still unclear yet. It has been reported that NOB shows various beneficial effects (Table 1), such as anticancer [5], anti-inflammation [1], antioxidation [6], antiinsulin resistance [7], antiosteoclastogenesis [8], immunomodulation [9], cardiovascular protection [10], and neuroprotection [11]. In this review article, we will discuss the pharmacological effects of NOB and its metabolites on diseases.

## 2. NOB Exhibits Protection in the Neural System

Accumulated amyloid *β* (A*β*) contributes to Alzheimer's disease (AD) pathogenesis. Impairments of learning and memory are the two main features of AD. It has been demonstrated that NOB may ameliorate such impairments in amyloid precursor protein transgenic mice, olfactory-bulbectomized mice, and* N*-methyl-d-aspartate (NMDA) receptor antagonist-treated mice. NOB also improves the aging cognitive impairment and reduces tau phosphorylation and oxidative stress in senescence-accelerated mouse prone 8 (SAMP8) mice [12] (Figure 3). Consistently, NOB may reduce the production of soluble A*β*_1-40_ in a triple transgenic mouse AD model (3XTg-AD), leading to amelioration of cognitive and memory impairment [13]. Chotosan (CTS) is a traditional Kampo prescription for chronic headache and recently used for ameliorating dementia. CTS-E, an ethyl acetate fraction of CTS, has been showed to protect against A*β*-induced neurotoxicity. NOB may exhibit neuroprotective effects against A*β*_1-42_ self-induced aggregation in PC12 cells [14]. Neprilysin (NEP) is one of the enzymes for degrading A*β*. NOB has been showed to upregulate the expression of NEP in a time and dose dependent manner in SK-N-SH cells [15] (Table 1). The beneficial effects of NOB on memory-improving in various animal models and against pathological characteristics of AD have been comprehensively reviewed [16].

**Figure 2:**
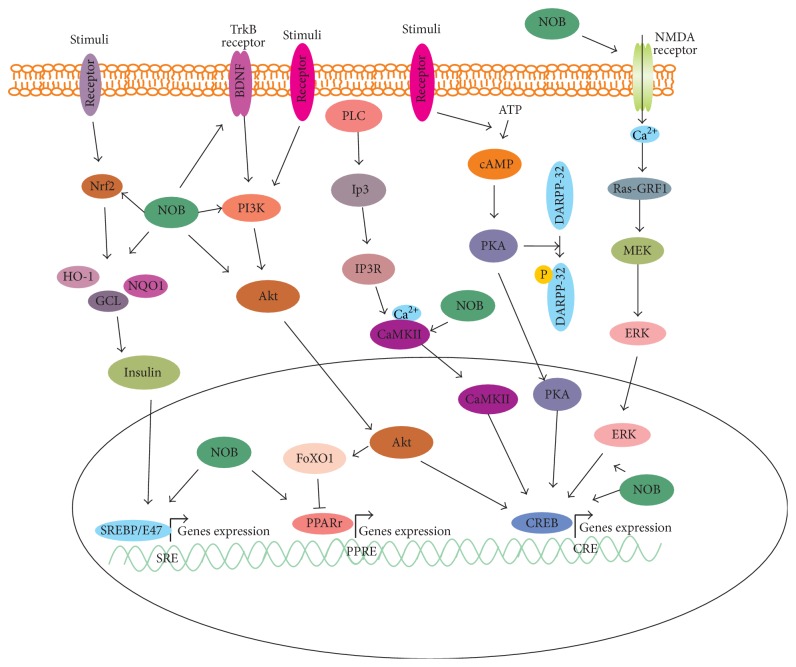
The possible signaling cascades of NOB in cells. NOB may upregulate the activities of NMDA receptor and ERK and enhance CREB transcriptional activity. NOB may restore the activity of DARPP-32 through modulation of PKA signaling pathway. Ca^2+^/CaMKII signaling is also enhanced by NOB, leading to upregulation of CREB transcription. In addition, NOB increases the activities of PI3K/Akt and BDNF/TrkB signaling but upregulates the expression of PPAR*γ*. However, it depends on different cell lines. Insulin sensitization has been elevated by NOB through Nrf2 signaling pathway.

The NMDA receptor is also essential for memory and learning (Figure 2). In PC12 cells, NOB has been reported to upregulate the mRNA expression of NMDA receptors subunits NR1, NR2A, and NR2B and c-FOS. NOB also improves learning impairment stimulated by NMDA receptor antagonist through activation of extracellular signal-mediated kinases. These activities might be associated with upregulation of CRE-dependent transcription linked to the activation of PKA/ERK/MEK/CREB signaling pathway [17], which is critical for memory and learning. NOB may potentiate upregulation of CRE-mediated transcription and facilitate PKA/ERK/CREB signaling in cultured hippocampal neurons [18] (Table 1). In PC12 cells, honeybee royal jelly upregulates the transcription activity mediated by CRE through an ERK-independent signaling pathway. However, coadministration of NOB and honeybee royal jelly may enhance CRE-mediated transcriptional activity in an ERK phosphorylation dependent manner [19].

**Figure 3:**
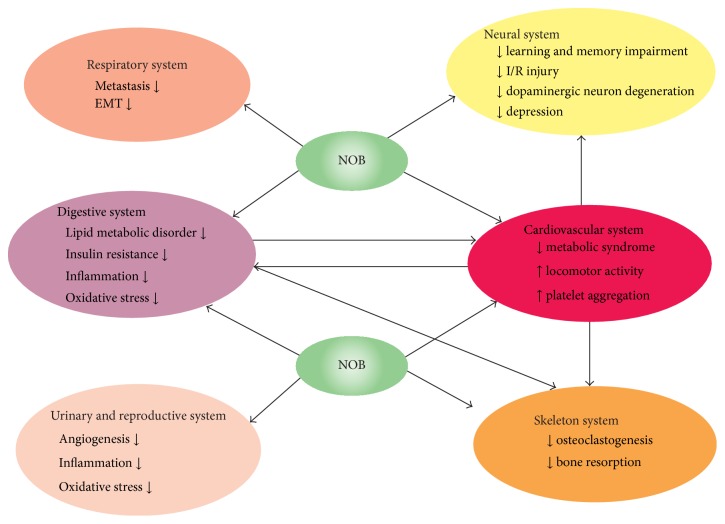
Summary of pharmacological effects of NOB on different systems. NOB may exhibit neuroprotective activity in attenuation of learning and memory impairment, amelioration of I/R injury, and decrease of dopaminergic neuron generation. In the cardiovascular system, NOB ameliorates metabolic syndrome, promotes locomotor activity, and inhibits platelet aggregation. In addition, NOB may rescue insulin resistance, restore lipid metabolic disorder, and downregulate inflammatory stress and oxidative stress in digestive system. The activities of antiangiogenesis and antimetastasis of NOB have been demonstrated in urinary, reproductive, and respiratory systems. NOB inhibits osteoclastogenesis and subsequently attenuates bone resorption, protecting skeleton homeostasis.

NOB may protect the brain against AD development through activation of CREB signaling pathway, which is involved in neuronal cells protection in ischemia-reperfusion (I/R) injury (Figure 3). NOB reduces the infarct volume, suppresses the brain edema, and inhibits cell death [20]. Akt/CREB signaling pathway contributes to neuronal survival and protection in the strategy of managing ischemic cerebral injury. NOB has been demonstrated to significantly upregulate the expression of CREB, Akt, BDNF, and Bcl-2, leading to amelioration of neurological deficits, brain edema, and brain blood barrier (BBB) permeability in rats [21] (Table 1).

Postischemic inflammation and oxidative stress play important roles in ischemic stroke pathogenesis. NOB has been showed to upregulate the expression of HO-1, Nrf2, GSH, and SOD1, while downregulating the expression of MMP-9, MDA, and NF-*κ*B, resulting in neuroprotection against brain edema and neurological deficits [11]. Oxidative stress may induce cell damage, resulting in pathological changes such as neurobiological disorder and AD. In hydrogen peroxide-treated HT22 cells, NOB may downregulate the phosphorylation levels of p38 and JNK and the expression of Bax and caspase-3, while upregulating the expression of Bcl-2, thus preventing cognitive impairment and protecting neurons from apoptosis [22]. In abnormal astrocytes, the overproduction of NO and cytokines may result in neurodegeneration. In rat primary astrocytes, NOB inhibits the expression of iNOS and decreases NO production via suppression of NF-*κ*B signaling and p38-MAPK phosphorylation [6].

Dopaminergic neuron degeneration in the substantia nigra is the critical feature in Parkinson's disease (PD) pathogenesis (Figure 3). In 1-methyl-4-phenylpyridinium (MPP^+^)-triggered rat PD, NOB inhibits the activation of microglia, preserves the expression of neurotrophic factor derived from the glial cells, and thus ameliorates dopaminergic neuron degeneration [23]. In 1-methyl-4-phenyl-1,2,3,6- tetrahydropyridine- (MPTP-) triggered mice PD, NOB may not block the degeneration of dopaminergic neurons but enhance the release of dopamine in striatum and hippocampal CA1. NOB restores the attenuated activity of Ca^2+^/calmodulin-dependent protein kinase II (CaMKII) autophosphorylation and cAMP-regulated phosphoprotein-32 (DARPP-32), leading to restoration of the decreased activity of tyrosine hydroxylase (TH) and rescue of cognitive and motor dysfunctions induced by MPTP [24].

Glioma is one of the most common tumors in primary central nervous system. NOB has been investigated to inhibit the cellular proliferation through suppression of Ras activity and MEK/ERK signaling pathway in a conventional PKC- and Ca^2+^-dependent manner in C6 glioma cells [25]. In human U87 and Hs683 glioma cell lines, NOB decreases cell viability and arrests at G_0_/G_1_ phase in cell cycle. These might be related to downregulation of cyclin D1, E2 promoter-binding factor 1, cyclin-dependent kinase (CDK) 2, CDK4, and phosphorylation levels of PKB, p38 MAPK, ERK, and JNK [26]. In SK-N-SH human neuroblastoma cells, NOB significantly reduces the expression of thioredoxin-interacting protein (TXNIP), which is one crucial factor for endoplasmic reticulum stress resulting in cell apoptosis [27].

Depression is an affective disorder or mood caused by various factors, including environmental or genetic factors. Upregulation of BDNF or activation of TrkB signaling pathways may reach antidepressant therapy. Five-week administration of NOB can significantly ameliorate chronic unpredictable mild stress- (CUMS-) induced deficits of BDNF, TrkB, and synapsin I in the hippocampus, resulting in an antidepressant-like response [28] (Figure 2). NOB may upregulate synaptic transmission to anti-immobility in mice in the forced swimming test (FST) and tail suspension test (TST). Furthermore, this antidepressant-like activity of NOB has been indicated to be associated with serotonergic, noradrenergic, and dopaminergic systems [29].

Deficiency of thyroid hormone may induce neural dysfunctions. The production of oligomycin-insensitive and oligomycin-sensitive ATP in the synaptic and nonsynaptic mitochondria of hippocampus in hypothyroid rats is decreased. These may be counteracted by NOB, which restores disordered mitochondrial metabolism through, at least partially, improving the activities of hexokinase and succinate dehydrogenase [30]. NOB has been demonstrated to stimulate the synthesis and secretion of catecholamine in bovine adrenal medulla through upregulation of phosphorylation at Ser19 and Ser40 in tyrosine hydroxylase, while downregulating catecholamine synthesis induced by acetylcholine [31]. The potential pharmacology of plant-derived flavonoids in the catecholamine system has been reviewed [32].

## 3. NOB Exhibits Protection in the Digestive System

Ammonia detoxification plays a critical role in body function, and it is also essential for physiological and nitrogen homeostasis. NOB has been demonstrated to be involved in the ammonia control across varying diets, regulating urea cycle activity through C/EBP-dependent regulatory mechanisms [33]. NOB regulates lipid and glucose metabolism, improving dyslipidemia, adiposity, hyperglycemia, and insulin resistance (Figure 3). NOB may decrease plasma triglyceride, body weight gain, and white adipose tissue (WAT) weight. In addition, NOB increases the expression of PPAR*α*, PPAR*γ*, fatty acid synthase, sterol regulatory element-binding protein-1c, stearoyl-CoA desaturase-1, carnitine palmitoyltransferase 1, adiponectin, and uncoupling protein-2 and decreases the expression of monocyte chemoattractant protein-1 and TNF*α* in WAT (Figure 2). Moreover, NOB upregulates the expression of glucose transporter-4 (GLUT4) and the phosphorylation of Akt and downregulates the degradation of I*κ*B*α* [34] (Table 1). GLUT4 activity is greatly dependent on PI3K signaling pathway. It has been demonstrated that NOB significantly promotes glucose uptake in 3T3-F442A adipocytes in a dose dependent manner through activation of PI3K/Akt1/2 and PKA/CREB signaling pathways [35].

To improve and prevent obesity and some related metabolic syndromes, NOB effectively inhibits the differentiation of 3T3-L1 preadipocytes into adipocytes. The underlying mechanisms might be associated with upregulation of cAMP elevator 3-isobutyl-1-methylxanthine (IBMX), insulin, and phosphorylation of signal transducer and activator of transcription (STAT) 5 and downregulation of PPAR*γ*2 and CREB phosphorylation [36]. NOB also processes less intracellular triglyceride, suppresses its accumulation, increases the secretion of an insulin-sensitizing factor, adiponectin, and decreases the secretion of insulin resistance factors, MCP-1 and resistin, in 3T3-L1 preadipocytes [7] (Table 1). The expression of stearoyl-CoA desaturase-1 (SCD1) is associated with improvement of glucose tolerance, decrease of plasma lipid level, and attenuation of obesity. NOB may significantly repress SCD1 mRNA expression in freshly isolated hepatocytes, resulting in reduction of hyperlipidemia and adiposity [37].

It has been demonstrated that NOB significantly reduces lipid accumulation, downregulates the activity of glycerol-3-phosphate dehydrogenase (GPDH), and attenuates the expression of adipogenic transcription factors, including peroxisome proliferator-activated receptors (PPAR*γ*) and CCAAT/enhancer binding protein (C/EBP*α*). Furthermore, NOB exhibits antiadipogenic activity through activation of AMP-activated protein kinase (AMPK) in 3T3-L1 cells [38]. The blood lipid lowing effects of NOB in HepG2 cells have been investigated that NOB may potently inhibit apoB secretion (IC_50_ = 29 *μ*M), cholesterol synthesis (IC_50_ = 68 *μ*M), and triglyceride synthesis (IC_50_ = 73 *μ*M), without affecting the activity of LDL-receptor (Table 1). The structure-activity study shows that full methoxylation in A-ring contributes to the potent inhibitory activity of NOB on the secretion of hepatic apoB [39]. The flavonoids including NOB from* Citrus* exhibit protection from aberrant lipid metabolism has been reviewed [40].

Chronic inflammatory disorder in the gastrointestinal tract is a main feature of inflammatory bowel disease. NOB may exhibit anti-inflammatory effects on TNBS-triggered colitis through downregulation of COX-2 and iNOS expression. NOB restores the barrier function impaired by TNBS through attenuation of Akt-NF-*κ*B-MLCK signaling pathway [41]. In IgE- and lipopolysaccharide- (LPS-) stimulated human intestinal mast cells, NOB may effectively downregulate the expression of CCL3, CCL4, CXCL8, IL-1*β*, and TNF*α* in a dose dependent manner. ERK1/2 signaling pathway is involved in NOB regulating effects on IgE stimulation, while NF-*κ*B signaling is involved in LPS stimulation [42].

In human gastric adenocarcinoma AGS cells, NOB downregulates the expression of MMP-2, MMP-9, c-Raf, Ras, Rac-1, RhoA, and Cdc42 through inhibition of PI3K/Akt signaling, focal adhesion kinase (FAK) activation, and NF-*κ*B signaling [5]. NOB has been reported to effectively inhibit the proliferation of p53-mutated SNU-16 human gastric cancer cells. NOB arrests cell cycle at sub-G_1_ phase, increases the expression of caspase-3 and caspase-9, the ratio of Bax/Bcl-2, and the degradation of poly (ADP-ribose) polymerase (PARP) protein, and promotes cell apoptosis. Additionally, NOB may synergize with 5-fluorouracil (5-FU) to exhibit an anticancer effect through two different mechanical pathways [43]. Pepsin, a digestive protease, is responsible for most digestion activities in the stomach. NOB may inhibit the activity of pepsin* in vitro*. The molecular interacting model has been theoretically simulated and docked by the software. It shows that NOB may spontaneously bind at the binding site of pepsin through electrostatic and hydrophobic forces [44].

Acute pancreatitis (AP) has been featured by oxidative stress, inflammation, and acinar cell damage. Intraperitoneal administration of NOB in C57BL/6 mice may ameliorate l-arginine-induced AP, which shows increased levels of plasma amylase, pancreatic myeloperoxidase, plasma proinflammatory factors, reactive oxygen species production, cell apoptosis, pancreatic necrosis, and the expression of p-p38MAPK and p-AKT [45]. It has been demonstrated that NOB may exhibit anti-inflammatory activity through downregulation of NF-*κ*B signaling pathway and the expression of iNOS. In hepatocytes, NOB suppresses the induction of NO with an IC_50_ value of 50 *μ*M [1]. Comparably, in LPS-induced mice RAW 264.7 macrophage cells, NOB shows an IC_50_ value of 27 *μ*M to suppress NO production [46]. Similarly, NOB at the dose of 100 *μ*M also inhibits LPS-induced secretion of proinflammatory cytokines, including IL-1*β*, TNF*α*, IL-6, and NO in BV2 microglia cells [47].

The* in vivo* and* in vitro* investigation of NOB on hepatic cancer cells has been performed.* In vitro*, NOB significantly inhibits cell proliferation, arrests cell cycle at G_2_ phase, downregulates Bcl-2 and COX-2 expression, and upregulates Bax and caspase-3 expression in SMMC-7721 cells. These results have been supported by the* in vivo* study in the H22 transplantable tumor [48]. Intestinal motility-related disorders may include increased or decreased motility. Investigations show that NOB may exhibit inhibitory effects on jejunal contractility in the high contractile states and stimulatory effects in the low contractile states. This might be applicable to the alternating management of type bowel hypo- and hypermotility [49].

## 4. NOB Exhibits Protection in the Cardiovascular and Blood Systems

Hyperglycemia triggered oxidative stress greatly contributes to cardiovascular dysfunctions. NOB has been demonstrated to ameliorate the oxidative stress, hemodynamic parameters, and vascular reactivity and downregulate the expression of MMP-2, MMP-9, and collagen in male Wistar rats [10] (Figure 3). In streptozotocin- (STZ-) induced C57BL mice diabetic cardiomyopathy, NOB significantly decreases the expression of TGF*β*1, fibronectin, CTGF, collagen 1*α*, and NADPH oxidase isoforms, including p22^phox^, p67^phox^, and p91^phox^. In addition, NOB also inhibits p38 MAPK/JNK and NF-*κ*B signaling pathway, leading to mitigation of interstitial fibrosis and cardiac dysfunction [50]. Insulin resistance contributes to lipid abnormalities including the accumulation of hepatic VLDL, leading to increased plasma concentration of apolipoprotein B100- (apoB100-) containing lipoprotein. The activity of NOB has been investigated to prevent dyslipidemia, insulin resistance, and atherogenesis. Research data show that NOB decreases the secretion of apoB100 through activation of MAPK^erk^, enhancement of LDLR expression, and attenuation of MTP activity and* DGAT1/2* expression. In addition, NOB decreases the availability of hepatic TG in a PPAR independent manner through upregulation of Pgc1*α* and Cpt1*α* and enhancement of *β*-oxidation [51].

NOB has been identified as a clock amplitude-enhancing factor through directly targeting retinoid acid receptor-related orphan receptors. In diet-induced obese mice, NOB, in a* Clock* gene dependent manner, dramatically ameliorates the metabolic syndrome, enhances energy expenditure, and augments locomotor activity, leading to mitigating metabolic disorders and improving circadian rhythms [52] (Figure 3). The abnormal proliferation and migration of vascular smooth muscle cells (VSMCs) contribute to neointimal hyperplasia development after vascular injury. In platelet-derived growth factor- (PDGF-) BB treated VSMCs, the proliferation, migration, the production of reactive oxygen species (ROS), ERK1/2 phosphorylation, and NF-*κ*B p65 nuclear translocation have been significantly increased. However, these detrimental responses may be inhibited by NOB [53].

Platelet activation plays an important role in CVDs. NOB inhibits platelet aggregation induced by collagen and arachidonic acid in the washed human platelets, but it does not inhibit the aggregation induced by thrombin and 9,11-dideoxy-11*α*,9*α*-epoxymethanoprostaglandin. The underlying mechanism may be associated with hydroxyl radical scavenge, inhibition of PLC*γ*2/PKC signaling, and suppression of MAPKs and Akt activation [54]. Active thrombin-activatable fibrinolysis inhibitor (TAFI) plays a critical role in balancing between fibrinolysis and coagulation. NOB has been demonstrated to exhibit anticoagulant activity and downregulate the expression of TAFI gene (*CPB2*) mRNA and antigen. In NOB-treated human hepatoma HepG2 cells, the amount of complex of AP-1 or c-Jun binding to the sequence at ~−119 bp to −99 bp within the* CPB2* promoter has been showed to be decreased [55].

In acute myeloid leukemia (AML) cells, NOB induces cell cycle arrest at the G_0_/G_1_ phase through downregulating ERK signaling pathway, increases cell apoptosis through activation of caspase-3, caspase-9, and caspase-8, and upregulates of MAPK signaling pathway in HL-60 cell line. Furthermore, NOB inhibits cell proliferation in various types of AML cell lines [56] (Table 1). Natural killer (NK) cells are the first guarder against virus-infected cells and cancer cells. KHYG-1 is an NK leukemia cell line. NOB may potentiate the cytolytic activity of KHYG-1 cells through dramatically enhancing the expression of granzyme B, which is partially regulated by p38 MAPK activity [57].

## 5. NOB Exhibits Protection in the Urinary and Reproductive Systems

NOB has been showed to lower the risk of prostate cancer (Figure 3). In PC-3 cells, NOB effectively reduces cell viability and attenuates the expression of VEGF and NF-*κ*B through downregulation of AKT/HIF-1*α* signaling pathway. In the less metastatic DU-145 cells, NOB decreases cMyc expression and p50 activity in the nuclei [58]. The effects of NOB on 2-amino-1-methyl-6-phenylimidazo [4,5-*b*]pyridine- (PhIP-) induced prostate carcinogenesis show that the levels of estrogen, leptin, and serum testosterone do not differ from those in the control group. However, the multiplicity and the incidence of adenocarcinomas in prostate in F344 rats have been reduced by NOB [59]. It has been demonstrated that NOB may exhibit the inhibitory effects against oxidative stress and apoptosis induced by cisplatin in acute renal injury [60].

In MDA-MB-231 human breast cancer cells, NOB downregulates the constitutive expressions of MMP-9 and CXCR4 with IC_50_ values of 24 *μ*M and 32 *μ*M, respectively, at transcriptional level through attenuation of MAPKs and NF-*κ*B signaling pathways, leading to effective inhibition of cancer cells metastasis and invasion [61]. In OVCAR-3 and A2780/CP70 ovarian cancer cells, NOB may significantly inhibit cell viability, proliferation, and VEGF protein secretion [62]. In HER2-positive SK-BR-3, hormone receptor-positive MCF7, and triple-negative MDA-MB-468 cell lines, NOB has been demonstrated to promote cell cycle arrest at G_0_/G_1_ phase through downregulation of ERK1/2 and cyclin D1 and upregulation of p21 in a dose and time dependent manner. In addition, NOB promotes cell apoptosis through decreasing the expression of Bcl-xL, Akt, and mTOR in MDA-MB-468 cells without changing Bax expressing profile [63]. At the dose of more than 40 *μ*M, NOB potently reduces human ovarian cancer cells viability. The possible mechanisms might be associated with decreasing the expression of the key mediators in angiogenesis, including HIF-1*α*, Akt, NF-*κ*B, and VEGF [64] (Table 1).

In human fetal membranes and myometrium taken after spontaneous preterm birth, NOB has been demonstrated to dramatically inhibit LPS-induced expression of proinflammatory cytokines, including IL-1*β*, TNF*α*, IL-8, and IL-6, and downregulate the expression of MMP-9 and COX-2 and decrease the secretion of pro-MMP-9 and PGE2 [65].

## 6. NOB Exhibits Protection in the Respiratory System

Hypoxia-induced epithelial-mesenchymal transition (EMT), an early step of tumor metastasis, has been showed to increase the expression of N-cadherin and vimentin and decrease the expression of E-cadherin. NOB has been identified as a Notch-1 inhibitor and to ameliorate hypoxia-induced EMT, as indicated by downregulation of Notch-1, Jagged 1/2, Hey-1, and Hes-1 in H1299 cells (Figure 3). In addition, NOB also promotes the expression of tumor suppressive factor miR-200b [66]. Transforming growth factor *β* (TGF*β*) is a stimulator of EMT, which is an important process in cancer metastasis. NOB has been demonstrated to inhibit TGF*β*1-stimulated EMT and downregulate the expression of p-Src, p-paxillin, p-FAK, MMP-2, MMP-9, slug, snail, ZEB1, and twist in lung adenocarcinoma H1299 and A549 cells. Additionally, NOB attenuates Smads transcriptional activity, and NOB does not change the phosphorylation levels and translocation of Smads stimulated by TGF*β*1. Overexpression of Smad3 abrogates the effects of NOB on EMT stimulated by TGF*β*1 [67].

Abnormal ion transport and epithelial damage greatly affect the structure of the airway surface liquid and airway mucus clearance. Studies show that NOB may promote the secretion of transepithelial Cl^−^ across bronchial epithelia through activation of cAMP/PKA- and adenylate cyclase-dependent signaling pathways and apical CFTR Cl^−^ channels in human bronchial epithelial cells (16HBE14o-) [68]. In nasopharyngeal carcinoma (NPC), NOB has been showed to inhibit the migration capacity of NPC-BM and HONE-1 cell lines. The potential mechanism might be related to inhibition of MMP-2 expression, NF-*κ*B, and AP-1 and enhancement of tissue inhibitor of metalloproteinase-2 (TIMP-2) [69].

## 7. NOB Exhibits Protection in the Skeleton System

Estrogen plays an important role in bone homeostasis. Postmenopausal women or ovariectomized (OVX) patients appear to show high risk of bone resorption. It has been demonstrated that NOB may prevent decrease of bone mass induced by estrogen deficiency and inhibit systemic bone resorption (Figure 3). The underlying mechanism may be associated with suppression of osteoclast formation induced by LPS and downregulation of osteoclastogenesis induced by receptor activator of NF-*κ*B ligands in RAW 264.7 [8]. NOB may suppress IL-1-induced bone resorption and osteoclast formation* in vitro*. Furthermore, NOB downregulates the expression of NF-*κ*B signaling, COX-2, and PGE2. In OVX mice, NOB may restore the bone mass and be beneficial to bone health [70] (Table 1). NOB has been demonstrated to significantly inhibit the motility, migration, and invasion of human osteosarcoma HOS and U2OS cells through downregulation of ERK/JNK signaling-mediated MMP-2 and MMP-9 expressions and inactivation of CREB, SP-1, and NF-*κ*B [71].

## 8. The Metabolites of Nobiletin

**Figure 4:**
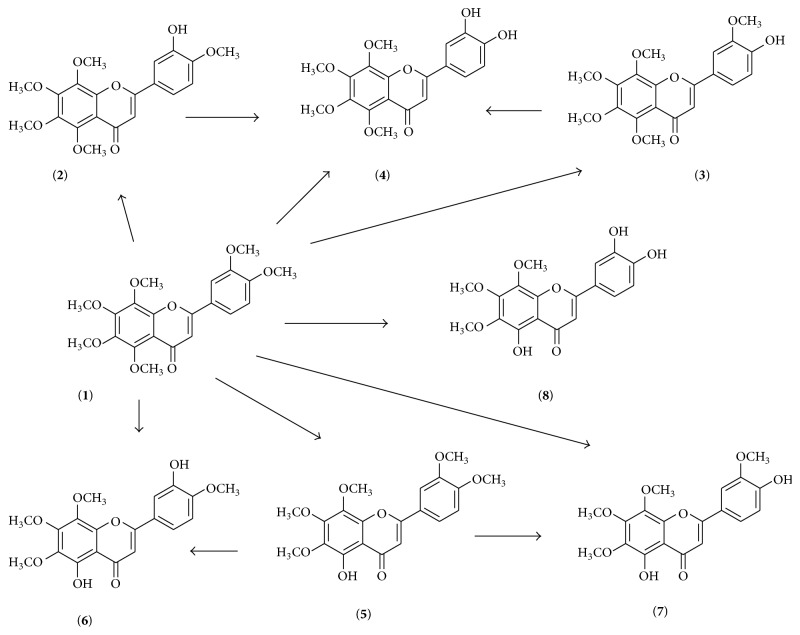
The structures of NOB metabolites, including NOB** (1)**, 3′-demethylnobiletin** (2)**, 4′-demethylnobiletin** (3)**, 3′,4′-didemethylnobiletin** (4)**, 5-demethylnobiletin** (5)**, 5,3′-didemethylnobiletin** (6)**, 5,4′-didemethylnobiletin** (7)**, and 5,3′,4′-tridemethylnobiletin** (8)**.

The natural occurring flavonoids have been demonstrated to be metabolized by P450 CYP1-enzyme, which leads to the activation of the metabolites, showing greater biological effects than their parent compounds. In MCF7 breast adenocarcinoma cells, the recombinant CYP1 enzymes convert NOB into one main metabolite* O*-demethylnobiletin [72]. Seven metabolites (Figure 4) including 3′-demethylnobiletin, 4′-demethylnobiletin, 3′,4′-didemethylnobiletin (DTF), 5-demethylnobiletin, 5,3′-didemethylnobiletin, 5,4′-didemethylnobiletin, and 5,3′,4′-tridemethylnobiletin have been identified as the major metabolites from the urine of mouse by employing the optimized HPLC method [73]. NOB is converted to 4′-demethylnobiletin, 6-demethylnobiletin, and 7-demethylnobiletin with a relative ratio of 1 : 4.1 : 0.5, respectively. CYP1A2 and CYP3A4 are the two key enzymes for regulating the oxidative demethylation of NOB in the A-ring and B-ring in human liver microsomes [74]. C-3′ and C-4′ positions are the primary sites for NOB biotransformation. The position and number of hydroxyl and methoxyl groups on the B-ring of NOB may significantly influence its metabolism and biological effects [75].

NOB has been demonstrated to exhibit significant antiproliferative, anti-inflammatory, and proapoptotic activities in the azoxymethane (AOM)/DSS-treated mice colon models. However, its colonic concentration is 20-fold lower than its three metabolites 3′-demethylnobiletin, 4′-demethylnobiletin, and DTF in the colonic mucosa. In addition, DTF exhibits the strongest anticancer activity. This indicates the important role of demethylation at C-3′ and C-4′ in inhibiting colon carcinogenesis [76]. DTF has showed antioxidative and anti-inflammatory activity. DTF exhibits neuroprotective functions through attenuation of NF-*κ*B signaling, decreasing the production of ROS, and upregulation of GCL and HO-1, which is independent of Nrf2. DTF also triggers the activation of ERK, Akt, and JNK signaling, but not p38 MAPK signaling pathway in PC12 cells [77]. DTF exhibits stronger antiatherogenic effects than NOB, as it indicated that DTF reduces Cu^2+^-induced LDL oxidation, inhibits the differentiation of monocyte into macrophage, and abrogates uptake of modified LDL by macrophage in THP-1 cells [78].

5-Demethylnobiletin at the dose of 10 *μ*M has been showed to cause 37% inhibition of SW620 cell viability. 5,3′-Didemethylnobiletin, 5,4′-didemethylnobiletin, and 5,3′,4′-tridemethylnobiletin show stronger inhibitory activity than 5-demethylnobiletin. The IC_50_ in SW620 is 0.12, 5.5, and 4.2 *μ*M, respectively. Analysis of structure-activity suggests that demethylation at C-3′, C-4′, or both C-3′ and C-4′ may enhance the inhibitory effects of 5-demethylnobiletin against colon cancer cells [79]. Similarly, these three metabolites show stronger inhibitory activities in the growth and colony formation than 5-demethylnobiletin in H1299 and H460 cells [80]. 5-Demethylnobiletin also exhibits antiatherogenic and hypolipidemic activities through induction of LDL-receptor gene expression and attenuation of acyl CoA:diacylglycerol acetyltransferase 2 expression in HepG2 cells [81]. In antibacterial assays, it has been demonstrated that demethylation at C-5 and C-4′ is essential [82].

Consistently, 4′-demethylnobiletin shows stronger bioactivities in anti-inflammation and anticancers. In LPS-treated RAW 264.7 macrophages, 4′-demethylnobiletin downregulates the expression of proinflammatory cytokines IL-1*β*, IL-6, PGE2, iNOS, and COX-2 and inhibits NF-*κ*B and AP-1 nuclear translocation, while upregulating the expression of transcription factor Nrf2 and its dependent genes HO-1 and NQO1 [83]. This is supported by the anti-inflammatory effects of 4′-demethylnobiletin on TPA-treated mice ear inflammation through inhibition of PI3K/Akt/ERK phosphorylation [84].

## 9. Miscellaneous Section

Mild inflammatory response is considered as a protective immune reaction. However, overwhelming immune response regulated by proinflammatory cytokines can be harmful. Endotoxin injection in mice introduces a septic shock model. NOB may significantly decrease the expression of proinflammatory cytokines IL-6 and TNF*α* in the early phase and HMGB1 in the late phase in the tissues of lung, liver, and kidney [9]. Synergized with sulforaphane, NOB exhibits a dramatic inhibitory effect on NO production and COX-2 and iNOS expression and promotes HO-1 protein expression in LPS-induced RAW 264.7 macrophage cells [85]. NOB also exhibits antiallergic effects. Study shows that histamine- or compound 48/80-triggered scratching behaviors may be inhibited by NOB, and the expressions of IL-4, TNF*α*, AP-1, NF-*κ*B, and p38 are also downregulated by NOB through inhibition of PKC signaling pathway in skin tissues and RBL-2H3 cells [86].

To investigate the anticancer activity of NOB, the alternations of gene expression in three organ-derived cell lines including HuH-7 human hepatocarcinoma cells, SK-N-SH human neuroblastoma cells, and 3Y1 rat fibroblasts are observed. NOB may induce the expression of endoplasmic reticulum stress-related genes* Trib3*,* Ddit3*, and* Asns*, attenuate cell cycle-responsive genes* E2f8*,* Ccna2*, and* Ccne2*, and decrease oxidative stress-mediated gene* Txnip *[87]. Angiogenesis plays a critical role in cancer development. NOB has been reported to exhibit antiangiogenic activity through modulation of the expression of VEGF-A dose dependently and promotion of G_0_/G_1_ phase accumulation in HUVEC cells [88].

The failure of chemotherapy treatment in many cancers is due to multidrug resistance (MDR). Investigation has showed that NOB may reverse ABCB1-regulated MDR without altering ABCB1 expression profiles through inhibition of ABCB1 transporter efflux function and suppression of Akt/ERK/Nrf2 signaling pathway relating to chemoresistance. Meanwhile, NOB exhibits a synergic activity with PTX to induce cell apoptosis and cell cycle arrest at G_2_/M phase in A2780/T cells [89]. Drugs interacting with beverages have been the focus in clinical application. Small molecule tyrosine kinase inhibitors (TKIs) attack cancer-specific targets and are the substrates of P-glycoprotein (P-gp) and Breast Cancer Resistance Protein (BCRP). Dasatinib is a dual BCRP and P-gp substrate. 14 natural polyphenols from beverages have been investigated for the efflux of dasatinib in LLC-PK1 cells and MDCK-II cells. NOB shows inhibiting dasatinib efflux mediated by both P-gp and BCRP. Furthermore, NOB exhibits greatest effects on increasing BCRP-regulated dasatinib uptake with IC_50_ value 1.04 *μ*M [90].

Endothelin-1 (ET) and stem cell factor (SCF) may trigger the normal human melanocytes to promote melanogenesis through activation of tyrosinase activity. Study shows that NOB significantly downregulates ET and SCF-induced gene expressions of tyrosinase, TRP1, PMEL, and MITF and decreases the phosphorylation of CREB, MEK/ERK1/2, and Raf-1, contributing to hypopigmentation [91]. MMPs are responsible for degradation of extracellular matrix proteins and collagen. NOB has been identified to a novel MMP-9 suppressor using MMP-9 reporter system through downregulation of p38 MAPK phosphorylation in human dermal fibroblasts stimulated by PMA [92].

## 10. Clinical Applications

NOB is one of the main flavonoids in* Citrus*. In hypercholesterolemic assays, daily administration of 30 mg tocotrienols and 270 mg citrus flavonoids for 4 weeks shows significant lowering of the levels of plasma total cholesterol, triglycerides, apoB100, and LDL cholesterol [93]. Similarly, consumption of orange juice (480 mL/day for one year) may reduce the concentration of total cholesterol, apoB100, and LDL cholesterol [94]. Studies demonstrated that administration of NOB-rich* C. reticulata* peel extract for 1 year exhibits preventive effects on the progression of the cognitive impairment in donepezil-preadministered AD patients without any side effects [95]. Unfortunately, the research on NOB clinical application is quite limited, which might be due to the uncertainty of molecular targets. More clinical trials of NOB and its metabolites are still needed.

## 11. Concluding Marks

The multifunctional physiological activities of NOB confer its possibility to become a promising candidate for developing as a therapeutic agent. The biological effects of NOB have been demonstrated to be associated with modulation of cellular signaling pathways, including cAMP/PKA/ERK/CREB, Ca^2+^/CaMKII, PI3K/Akt1/2, and NF-*κ*B, as well as its capacity of regulating specific genes expression. However, the underlying molecular mechanisms are still unclear. This might be due to the uncertainty of target receptor molecules. Although the effects of NOB* in vitro* have been well clarified using different cultured cells, the effects* in vivo* are still unclear. The protective effects of NOB should be examined using various animal models, and systemic data for considerable analysis are needed for more conclusive information and add to the knowledge about the pharmacological activities of NOB in diseases.

## Figures and Tables

**Table 1:** The biological effects of NOB on different cell types/animals.

Cell types/animals	Biological functions	Ref
3T3-L1 preadipocytes	Differentiation↑, IBMX↑, insulin↑, STAT 5↑, PPAR*γ*2↓, p-CREB↓	[36]
	Intracellular triglyceride↓, adiponectin↑, MCP-1↓, resistin↓	[7]
	Lipid accumulation↓, GPDH↓, PPAR*γ*↓, C/EBP*α*↓, AMPK↑	[38]

3T3-F442A adipocytes	Glucose uptake↑, PI3K/Akt1/2↑, PKA/CREB signaling↑	[35]

3XTg-AD mice model	Soluble A*β*_1-40_ production↓	[13]

A2780/T cells	ABCB1 transporter efflux↓, Akt/ERK/Nrf2↓	[89]

AGS cells	MMP-2↓, MMP-9↓, c-Raf↓, Ras↓, Rac-1↓, RhoA↓, Cdc42↓, PI3K/Akt signaling↓, FAK activation↓, NF-*κ*B signaling↓	[5]

BV2 microglia cells	IL-1*β*↓, TNF*α*↓, IL-6↓, NO↓	[47]

C6 glioma cells	cellular proliferation↓, Ras activity↓, MEK/ERK signaling↓	[25]

CUMS-induced rats	BDNF↑, TrkB↑, synapsin I↑	[28]

Diet-induced obese mice	Energy expenditure↑, locomotor activity↑, metabolic disorders↓, circadian rhythms↑	[52]

DU-145 cells	cMyc↓, p50↓	[58]

Freshly isolated hepatocytes	SCD1 mRNA expression↓, hyperlipidemia↓, adiposity↓	[37]

H1299 cells	NOTCH-1↓, Jagged 1/2↓, Hey-1↓, Hes-1↓	[66]

H1299, A549 cells	EMT↓, p-Src↓, p-paxillin↓, p-FAK↓, MMP-2↓, MMP-9↓, slug↓, snail↓, ZEB1↓, twist↓	[67]

HepG2 cells	ApoB secretion (IC_50_ = 29 *μ*M)↓, cholesterol synthesis (IC_50_ = 68 *μ*M)↓, triglyceride synthesis (IC_50_ = 73 *μ*M)↓	[39]
	Dyslipidemia↓, insulin resistance↓, atherogenesis↓, apoB100↓, MAPK^erk^↑, LDLR↑, MTP↓, *DGAT1/2*↓, Pgc1*α*↑, Cpt1*α*↑, *β*-oxidation↑	[51]

Hepatocytes	NO (IC_50_ = 50 *μ*M)↓	[1]

HFD-induced obese mice	Plasma triglyceride↓, body weight gain↓, WAT weight↓, PPAR*α*↑, PPAR*γ*↑, fatty acid synthase↑, sterol regulatory element-binding protein-1c↑, stearoyl-CoA desaturase-1↑, adiponectin↑, carnitine palmitoyltransferase 1↑, uncoupling protein-2↑, monocyte chemoattractant protein-1↓, TNF*α*↓, GLUT4↑, p-Akt↑, I*κ*B*α* degradation↓	[34]

HL-60 cell line	ERK↓, caspase-3↑, caspase-9↑, caspase-8↑, MAPK↑	[56]

HOS and U2OS cells	ERK/JNK↓, MMP-2↓, MMP-9↓, CREB↓, SP-1↓, NF-*κ*B↓	[71]

HT22 cells	p-p38↓, p-JNK↓, Bax↓, caspase-3↓, Bcl-2↑, cognitive impairment↓, apoptosis↓	[22]

HuH-7, SK-N-SH, 3Y1	*Trib3* ↓, *Ddit3*↓, *Asns*↓, *E2f8*↓, *Ccna2*↓, *Ccne2*↓, *Txnip*↓	[87]

Human intestinal mast cells	CCL3↓, CCL4↓, CXCL8↓, IL-1*β*↓, TNF*α*↓	[42]

Human ovarian cancer cells	HIF-1*α*↓, Akt↓, NF-*κ*B↓, VEGF↓	[64]

Human fetal membranes and myometrium	IL-1*β*↓, TNF*α*↓, IL-8↓, IL-6↓, MMP-9↓, COX-2↓, pro-MMP-9↓, PGE2↓	[65]

Human dermal fibroblasts	MMP-9↓, p-p38 MAPK↓	[92]

L-Arginine-induced AP	Plasma amylase↓, pancreatic myeloperoxidase↓, plasma proinflammatory factors↓, reactive oxygen species production↓, cell apoptosis↓, pancreatic necrosis↓, p-p38MAPK↓, p-AKT↓	[45]

MDA-MB-231 cells	MMP-9 (IC_50_ = 24 *μ*M)↓, CXCR4 (IC_50_ = 32 *μ*M)↓, MAPKs↓, NF-*κ*B↓	[61]

MDA-MB-468 cells	ERK1/2↓, cyclin D1↓, p21↑, Bcl-xL↓, Akt↓, mTOR↓	[63]

MPP^+^-triggered rat PD	Microglia activation↓, dopaminergic neuron degeneration↓	[23]

MPTP-triggered mice PD	Dopamine release↑, Ca^2+^/CaMKII autophosphorylation↑, DARPP-32↑, TH↑, cognitive and motor functions↑	[24]

NK leukemia cell	Granzyme B↑, p38 MAPK↑, cytolytic activity of KHYG-1 cells↑	[57]

NPC-BM, HONE-1 cells	MMP-2↓, NF-*κ*B↓, AP-1↓, TIMP-2↑	[69]

Normal human melanocytes	Tyrosinase↓, TRP1↓, PMEL↓, MITF↓, p-CREB↓, p-MEK/p-ERK1/2↓, p-Raf-1↓	[91]

OVCAR-3, A2780/CP70 cells	Cell Viability↓, proliferation↓, VEGF↓	[62]

OVX mice	Bone resorption↓, osteoclast formation↓, NF-*κ*B↓, COX-2↓, PGE2↓	[70]

P53-mutated SNU-16 human gastric cancer cells	Proliferation↓, caspase-3↑, caspase-9↑, Bax/Bcl-2 ratio↑, PARP degradation↑, apoptosis↑	[43]

PC12 cells	A*β*_1-42_ self-induced aggregation↓	[14]
	NR1↑, NR2A↑, NR2B↑, c-FOS↑, learning impairment↓, CRE-dependent transcription↑, PKA/ERK/MEK/CREB↑	[17]

PC-3 cells	Viability↓, VEGF↓, NF-*κ*B↓, AKT/HIF-1*α*↓	[58]

PDGF-BB treated VSMCs	Proliferation↓, migration↓, ROS↓, p-ERK1/2↓, NF-*κ*B p65 nuclear translocation↓	[53]

Postischemic	HO-1↑, Nrf2↑, GSH↑, SOD1↑, MMP-9↓, MDA↓, NF-*κ*B↓, neurological deficits↓, brain edema↓	[11]

Rats	Hexokinase↑, succinate dehydrogenase↑, mitochondrial metabolism↑	[30]

Rat I/R injury	Infarct volume↓, brain edema↓, cell death↓	[20]
	CREB↑, Akt↑, BDNF↑, Bcl-2↑, neurological deficits↓, brain edema↓, BBB permeability↓	[21]

Rat primary astrocytes	iNOS↓, NO production↓, NF-*κ*B↓, p38-MAPK phosphorylation↓	[6]

RAW 264.7 cells	NO (IC_50_ = 27 *μ*M)↓	[46]
	Bone resorption↓, osteoclast formation↓, osteoclastogenesis↓	[8]

RBL-2H3 cells	IL-4↓, TNF*α*↓, AP-1↓, NF-*κ*B↓, p38↓, PKC↓	[86]

SAMP8 mice model	Aging cognitive impairment↓, tau phosphorylation↓, oxidative stress↓	[12]

SK-N-SH cells	NEP↑	[15]
	TXNIP↓	[27]

SMMC-7721 cells	Proliferation↓, Bcl-2↓, COX-2↓, Bax↑, caspase-3↑	[48]

STZ-induced C57BL mice	TGF*β*1↓, fibronectin↓, CTGF↓, collagen 1*α*↓, p22^phox^↓, p67^phox^↓, p91^phox^↓, p38 MAPK/JNK↓, NF-*κ*B↓	[50]

STZ-induced rats	Oxidative stress↓, hemodynamic parameters↓, vascular reactivity↓, MMP-2↓, MMP-9↓, collagen↓	[10]

TNBS-triggered colitis	COX-2↓, iNOS↓, Akt-NF-*κ*B-MLCK signaling↓	[41]

U87, Hs683 glioma cells	Cell viability↓, cyclin D1↓, E2 promoter-binding factor 1↓, CDK2↓, CDK4↓, p-PKB↓, p-p38 MAPK↓, p-ERK↓, p-JNK↓	[26]

## References

[1] Yoshigai E., Machida T., Okuyama T. (2013). Citrus nobiletin suppresses inducible nitric oxide synthase gene expression in interleukin-1*β*-treated hepatocytes. *Biochemical and Biophysical Research Communications*.

[2] Noguchi S., Atsumi H., Iwao Y., Kan T., Itai S. (2016). Nobiletin: a citrus flavonoid displaying potent physiological activity. *Acta Crystallographica Section C: Structural Chemistry*.

[3] Singh S. P., Wahajuddin, Tewari D., Patel K., Jain G. K. (2011). Permeability determination and pharmacokinetic study of nobiletin in rat plasma and brain by validated high-performance liquid chromatography method. *Fitoterapia*.

[4] Kimura O., Ohta C., Koga N., Haraguchi K., Kato Y., Endo T. (2014). Carrier-mediated uptake of nobiletin, a citrus polymethoxyflavonoid, in human intestinal Caco-2 cells. *Food Chemistry*.

[5] Lee Y.-C., Cheng T.-H., Lee J.-S. (2011). Nobiletin, a citrus flavonoid, suppresses invasion and migration involving FAK/PI3K/Akt and small GTPase signals in human gastric adenocarcinoma AGS cells. *Molecular and Cellular Biochemistry*.

[6] Ihara H., Yamamoto H., Ida T. (2012). Inhibition of nitric oxide production and inducible nitric oxide synthase expression by a polymethoxyflavone from young fruits of *Citrus unshiu* in rat primary astrocytes. *Bioscience, Biotechnology and Biochemistry*.

[7] Miyata Y., Tanaka H., Shimada A. (2011). Regulation of adipocytokine secretion and adipocyte hypertrophy by polymethoxyflavonoids, nobiletin and tangeretin. *Life Sciences*.

[8] Tominari T., Hirata M., Matsumoto C., Inada M., Miyaura C. (2012). Polymethoxy flavonoids, nobiletin and tangeretin, prevent lipopolysaccharide-induced inflammatory bone loss in an experimental model for periodontitis. *Journal of Pharmacological Sciences*.

[9] Li W., Wang X., Niu X. (2016). Protective effects of nobiletin against endotoxic shock in mice through inhibiting TNF-*α*, IL-6, and HMGB1 and regulating NF-*κ*B pathway. *Inflammation*.

[10] Parkar N. A., Bhatt L. K., Addepalli V. (2016). Efficacy of nobiletin, a citrus flavonoid, in the treatment of the cardiovascular dysfunction of diabetes in rats. *Food & Function*.

[11] Zhang L., Zhang X., Zhang C. (2016). Nobiletin promotes antioxidant and anti-inflammatory responses and elicits protection against ischemic stroke in vivo. *Brain Research*.

[12] Nakajima A., Aoyama Y., Nguyen T.-T. L. (2013). Nobiletin, a citrus flavonoid, ameliorates cognitive impairment, oxidative burden, and hyperphosphorylation of tau in senescence-accelerated mouse. *Behavioural Brain Research*.

[13] Nakajima A., Aoyama Y., Shin E.-J. (2015). Nobiletin, a citrus flavonoid, improves cognitive impairment and reduces soluble A*β* levels in a triple transgenic mouse model of Alzheimer's disease (3XTg-AD). *Behavioural Brain Research*.

[14] Wei M., Chen L., Liu J., Zhao J., Liu W., Feng F. (2016). Protective effects of a Chotosan fraction and its active components on *β*-amyloid-induced neurotoxicity. *Neuroscience Letters*.

[15] Fujiwara H., Kimura J., Sakamoto M. (2014). Nobiletin, a flavone from *Citrus depressa*, induces gene expression and increases the protein level and activity of neprilysin in SK-N-SH cells. *Canadian Journal of Physiology and Pharmacology*.

[16] Nakajima A., Ohizumi Y., Yamada K. (2014). Anti-dementia activity of nobiletin, a citrus flavonoid: a review of animal studies. *Clinical Psychopharmacology and Neuroscience*.

[17] Kimura J., Nemoto K., Degawa M. (2014). Upregulation of N-methyl-D-aspartate receptor subunits and c-Fos expressing genes in PC12D cells by nobiletin. *Biological & Pharmaceutical Bulletin*.

[18] Kawahata I., Yoshida M., Sun W. (2013). Potent activity of nobiletin-rich *Citrus reticulata* peel extract to facilitate cAMP/PKA/ERK/CREB signaling associated with learning and memory in cultured hippocampal neurons: identification of the substances responsible for the pharmacological action. *Journal of Neural Transmission*.

[19] Fujiwara H., Kogure A., Sakamoto M. (2011). Honeybee royal jelly and nobiletin stimulate CRE-mediated transcription in ERK-independent and -dependent fashions, respectively, in PC12D cells. *Journal of Pharmacological Sciences*.

[20] Yasuda N., Ishii T., Oyama D. (2014). Neuroprotective effect of nobiletin on cerebral ischemia-reperfusion injury in transient middle cerebral artery-occluded rats. *Brain Research*.

[21] Zhang L., Zhao H., Zhang X. (2013). Nobiletin protects against cerebral ischemia via activating the p-Akt, p-CREB, BDNF and Bcl-2 pathway and ameliorating BBB permeability in rat. *Brain Research Bulletin*.

[22] Cho H., Jung S., Lee G., Cho J., Choi I. (2015). Neuroprotective effect of Citrus unshiu immature peel and nobiletin inhibiting hydrogen peroxide-induced oxidative stress in HT22 murine hippocampal neuronal cells. *Pharmacognosy Magazine*.

[23] Jeong K. H., Jeon M.-T., Kim H. D. (2015). Nobiletin protects dopaminergic neurons in the 1-methyl-4-phenylpyridinium-treated rat model of Parkinson's disease. *Journal of Medicinal Food*.

[24] Yabuki Y., Ohizumi Y., Yokosuka A., Mimaki Y., Fukunaga K. (2014). Nobiletin treatment improves motor and cognitive deficits seen in MPTP-induced parkinson model mice. *Neuroscience*.

[25] Aoki K., Yokosuka A., Mimaki Y., Fukunaga K., Yamakuni T. (2013). Nobiletin induces inhibitions of Ras activity and mitogen-activated protein kinase kinase/extracellular signal-regulated kinase signaling to suppress cell proliferation in C6 rat glioma cells. *Biological & Pharmaceutical Bulletin*.

[26] Lien L.-M., Wang M.-J., Chen R.-J. (2016). Nobiletin, a polymethoxylated flavone, inhibits glioma cell growth and migration via arresting cell cycle and suppressing MAPK and Akt Pathways. *Phytotherapy Research*.

[27] Ikeda A., Nemoto K., Yoshida C. (2013). Suppressive effect of nobiletin, a citrus polymethoxyflavonoid that downregulates thioredoxin-interacting protein expression, on tunicamycin-induced apoptosis in SK-N-SH human neuroblastoma cells. *Neuroscience Letters*.

[28] Li J., Zhou Y., Liu B.-B. (2013). Nobiletin ameliorates the deficits in hippocampal BDNF, TrkB, and synapsin I induced by chronic unpredictable mild stress. *Evidence-Based Complementary and Alternative Medicine*.

[29] Yi L.-T., Xu H.-L., Feng J., Zhan X., Zhou L.-P., Cui C.-C. (2011). Involvement of monoaminergic systems in the antidepressant-like effect of nobiletin. *Physiology & Behavior*.

[30] Jojua N., Sharikadze N., Zhuravliova E., Zaalishvili E., Mikeladze D. G. (2015). Nobiletin restores impaired hippocampal mitochondrial bioenergetics in hypothyroidism through activation of matrix substrate-level phosphorylation. *Nutritional Neuroscience*.

[31] Zhang H., Yanagihara N., Toyohira Y. (2014). Stimulatory effect of nobiletin, a citrus polymethoxy flavone, on catecholamine synthesis through Ser19 and Ser40 phosphorylation of tyrosine hydroxylase in cultured bovine adrenal medullary cells. *Naunyn-Schmiedeberg's Archives of Pharmacology*.

[32] Yanagihara N., Zhang H., Toyohira Y. (2014). New insights into the pharmacological potential of plant flavonoids in the catecholamine system. *Journal of Pharmacological Sciences*.

[33] Nohara K., Shin Y., Park N. (2015). Ammonia-lowering activities and carbamoyl phosphate synthetase 1 (Cps1) induction mechanism of a natural flavonoid. *Nutrition & Metabolism*.

[34] Lee Y.-S., Cha B.-Y., Choi S.-S. (2013). Nobiletin improves obesity and insulin resistance in high-fat diet-induced obese mice. *Journal of Nutritional Biochemistry*.

[35] Onda K., Horike N., Suzuki T.-I., Hirano T. (2013). Polymethoxyflavonoids tangeretin and nobiletin increase glucose uptake in murine adipocytes. *Phytotherapy Research*.

[36] Kanda K., Nishi K., Kadota A., Nishimoto S., Liu M.-C., Sugahara T. (2012). Nobiletin suppresses adipocyte differentiation of 3T3-L1 cells by an insulin and IBMX mixture induction. *Biochimica et Biophysica Acta—General Subjects*.

[37] Nichols L. A., Jackson D. E., Manthey J. A., Shukla S. D., Holland L. J. (2011). Citrus flavonoids repress the mRNA for stearoyl-CoA desaturase, a key enzyme in lipid synthesis and obesity control, in rat primary hepatocytes. *Lipids in Health and Disease*.

[38] Choi Y., Kim Y., Ham H., Park Y., Jeong H.-S., Lee J. (2011). Nobiletin suppresses adipogenesis by regulating the expression of adipogenic transcription factors and the activation of AMP-activated protein kinase (AMPK). *Journal of Agricultural and Food Chemistry*.

[39] Lin Y., Vermeer M. A., Bos W. (2011). Molecular structures of citrus flavonoids determine their effects on lipid metabolism in HepG2 cells by primarily suppressing apoB secretion. *Journal of Agricultural and Food Chemistry*.

[40] Assini J. M., Mulvihill E. E., Huff M. W. (2013). Citrus flavonoids and lipid metabolism. *Current Opinion in Lipidology*.

[41] Xiong Y., Chen D., Yu C. (2015). Citrus nobiletin ameliorates experimental colitis by reducing inflammation and restoring impaired intestinal barrier function. *Molecular Nutrition & Food Research*.

[42] Hagenlocher Y., Feilhauer K., Schäffer M., Bischoff S. C., Lorentz A. (2016). Citrus peel polymethoxyflavones nobiletin and tangeretin suppress LPS- and IgE-mediated activation of human intestinal mast cells. *European Journal of Nutrition*.

[43] Moon J. Y., Cho M., Ahn K. S., Cho S. K. (2013). Nobiletin induces apoptosis and potentiates the effects of the anticancer drug 5-fluorouracil in p53-mutated SNU-16 human gastric cancer cells. *Nutrition and Cancer*.

[44] Zeng H.-J., Qi T., Yang R., You J., Qu L.-B. (2014). Spectroscopy and molecular docking study on the interaction behavior between nobiletin and pepsin. *Journal of Fluorescence*.

[45] Liu B., Huang J., Zhang B. (2016). Nobiletin protects against murine l-arginine-induced acute pancreatitis in association with downregulating p38MAPK and AKT. *Biomedicine & Pharmacotherapy*.

[46] Choi S.-Y., Ko H.-C., Ko S.-Y. (2007). Correlation between flavonoid content and the NO production inhibitory activity of peel extracts from various citrus fruits. *Biological and Pharmaceutical Bulletin*.

[47] Ho S.-C., Kuo C.-T. (2014). Hesperidin, nobiletin, and tangeretin are collectively responsible for the anti-neuroinflammatory capacity of tangerine peel (*Citri reticulatae* pericarpium). *Food and Chemical Toxicology*.

[48] Ma X., Jin S., Zhang Y., Wan L., Zhao Y., Zhou L. (2014). Inhibitory effects of nobiletin on hepatocellular carcinoma in vitro and in vivo. *Phytotherapy Research*.

[49] Xiong Y.-J., Chen D.-P., Lv B.-C., Liu F.-F., Wang L., Lin Y. (2014). Characteristics of nobiletin-induced effects on jejunal contractility. *Fitoterapia*.

[50] Zhang N., Yang Z., Xiang S. (2016). Nobiletin attenuates cardiac dysfunction, oxidative stress, and inflammatory in streptozotocin: induced diabetic cardiomyopathy. *Molecular and Cellular Biochemistry*.

[51] Mulvihill E. E., Assini J. M., Lee J. K. (2011). Nobiletin attenuates VLDL overproduction, dyslipidemia, and atherosclerosis in mice with diet-induced insulin resistance. *Diabetes*.

[52] He B., Nohara K., Park N. (2016). The small molecule nobiletin targets the molecular oscillator to enhance circadian rhythms and protect against metabolic syndrome. *Cell Metabolism*.

[53] Guan S., Tang Q., Liu W., Zhu R., Li B. (2014). Nobiletin inhibits PDGF-BB-induced vascular smooth muscle cell proliferation and migration and attenuates neointimal hyperplasia in a rat carotid artery injury model. *Drug Development Research*.

[54] Lu W.-J., Lin K.-C., Liu C.-P. (2016). Prevention of arterial thrombosis by nobiletin: *in vitro* and *in vivo* studies. *Journal of Nutritional Biochemistry*.

[55] Takada K., Seike T., Sasaki T., Masuda Y., Ito A., Ishii H. (2013). Nobiletin, a polymethoxyflavone in citrus fruits, reduces TAFI expression in HepG2 cells through transcriptional inhibition. *Thrombosis and Haemostasis*.

[56] Hsiao P.-C., Lee W.-J., Yang S.-F. (2014). Nobiletin suppresses the proliferation and induces apoptosis involving MAPKs and caspase-8/-9/-3 signals in human acute myeloid leukemia cells. *Tumor Biology*.

[57] Saito T., Abe D., Nogata Y. (2015). Polymethoxylated flavones potentiate the cytolytic activity of NK leukemia cell line KHYG-1 via enhanced expression of granzyme B. *Biochemical and Biophysical Research Communications*.

[58] Chen J., Creed A., Chen A. Y. (2014). Nobiletin suppresses cell viability through AKT pathways in PC-3 and DU-145 prostate cancer cells. *BMC Pharmacology & Toxicology*.

[59] Tang M. X., Ogawa K., Asamoto M. (2011). Effects of nobiletin on PhIP-induced prostate and colon carcinogenesis in F344 rats. *Nutrition and Cancer*.

[60] Malik S., Bhatia J., Suchal K., Gamad N., Dinda A. K., Gupta Y. K. (2015). Nobiletin ameliorates cisplatin-induced acute kidney injury due to its anti-oxidant, anti-inflammatory and anti-apoptotic effects. *Experimental and Toxicologic Pathology*.

[61] Ahn K. S., Baek S. H., Kim S.-M. (2012). Antimetastatic effect of nobiletin through the down-regulation of CXC chemokine receptor type 4 and matrix metallopeptidase-9. *Pharmaceutical Biology*.

[62] He Z., Li B., Rankin G. O., Rojanasakul Y., Chen Y. C. (2015). Selecting bioactive phenolic compounds as potential agents to inhibit proliferation and VEGF expression in human ovarian cancer cells. *Oncology Letters*.

[63] Chen C., Ono M., Takeshima M., Nakano S. (2014). Antiproliferative and apoptosis-inducing activity of nobiletin against three subtypes of human breast cancer cell lines. *Anticancer Research*.

[64] Chen J., Chen A. Y., Huang H. (2015). The flavonoid nobiletin inhibits tumor growth and angiogenesis of ovarian cancers via the Akt pathway. *International Journal of Oncology*.

[65] Morwood C. J., Lappas M. (2014). The citrus flavone nobiletin reduces pro-inflammatory and pro-labour mediators in fetal membranes and myometrium: implications for preterm birth. *PLoS ONE*.

[66] Gao X.-J., Liu J.-W., Zhang Q.-G., Zhang J.-J., Xu H.-T., Liu H.-J. (2015). Nobiletin inhibited hypoxia-induced epithelial-mesenchymal transition of lung cancer cells by inactivating of Notch-1 signaling and switching on miR-200b. *Pharmazie*.

[67] Da C., Liu Y., Zhan Y., Liu K., Wang R. (2016). Nobiletin inhibits epithelial-mesenchymal transition of human non-small cell lung cancer cells by antagonizing the TGF-*β*1/Smad3 signaling pathway. *Oncology Reports*.

[68] Hao Y., Cheung C. S. T., Yip W. C. Y., Ko W.-H. (2015). Nobiletin stimulates chloride secretion in human bronchial epithelia via a cAMP/PKA-dependent pathway. *Cellular Physiology and Biochemistry*.

[69] Chien S.-Y., Hsieh M.-J., Chen C.-J., Yang S.-F., Chen M.-K. (2015). Nobiletin inhibits invasion and migration of human nasopharyngeal carcinoma cell lines by involving ERK1/2 and transcriptional inhibition of MMP-2. *Expert Opinion on Therapeutic Targets*.

[70] Harada S., Tominari T., Matsumoto C. (2011). Nobiletin, a polymethoxy flavonoid, suppresses bone resorption by inhibiting NF*κ*B-dependent prostaglandin E synthesis in osteoblasts and prevents bone loss due to estrogen deficiency. *Journal of Pharmacological Sciences*.

[71] Cheng H. L., Hsieh M. J., Yang J. S. (2016). Nobiletin inhibits human osteosarcoma cells metastasis by blocking ERK and JNK-mediated MMPs expression. *Oncotarget*.

[72] Surichan S., Androutsopoulos V. P., Sifakis S. (2012). Bioactivation of the citrus flavonoid nobiletin by CYP1 enzymes in MCF7 breast adenocarcinoma cells. *Food and Chemical Toxicology*.

[73] Zheng J., Bi J., Johnson D. (2015). Analysis of 10 metabolites of polymethoxyflavones with high sensitivity by electrochemical detection in high-performance liquid chromatography. *Journal of Agricultural and Food Chemistry*.

[74] Koga N., Ohta C., Kato Y. (2011). In vitro metabolism of nobiletin, a polymethoxy-flavonoid, by human liver microsomes and cytochrome P450. *Xenobiotica*.

[75] Li S., Sang S., Pan M.-H. (2007). Anti-inflammatory property of the urinary metabolites of nobiletin in mouse. *Bioorganic & Medicinal Chemistry Letters*.

[76] Wu X., Song M., Wang M. (2015). Chemopreventive effects of nobiletin and its colonic metabolites on colon carcinogenesis. *Molecular Nutrition & Food Research*.

[77] Su J.-D., Yen J.-H., Li S. (2012). 3′,4′-Didemethylnobiletin induces phase II detoxification gene expression and modulates PI3K/Akt signaling in PC12 cells. *Free Radical Biology & Medicine*.

[78] Lo Y.-H., Pan M.-H., Li S. (2010). Nobiletin metabolite, 3′,4′-dihydroxy-5,6,7,8-tetramethoxyflavone, inhibits LDL oxidation and down-regulates scavenger receptor expression and activity in THP-1 cells. *Biochimica et Biophysica Acta—Molecular and Cell Biology of Lipids*.

[79] Zheng J., Song M., Dong P. (2013). Identification of novel bioactive metabolites of 5-demethylnobiletin in mice. *Molecular Nutrition and Food Research*.

[80] Song M., Charoensinphon N., Wu X. (2016). Inhibitory effects of metabolites of 5-demethylnobiletin on human nonsmall cell lung cancer cells. *Journal of Agricultural and Food Chemistry*.

[81] Yen J.-H., Weng C.-Y., Li S. (2011). Citrus flavonoid 5-demethylnobiletin suppresses scavenger receptor expression in THP-1 cells and alters lipid homeostasis in HepG2 liver cells. *Molecular Nutrition & Food Research*.

[82] Wu T., Zang X., He M., Pan S., Xu X. (2013). Structure-activity relationship of flavonoids on their anti—*Escherichia coli* activity and inhibition of DNA gyrase. *Journal of Agricultural and Food Chemistry*.

[83] Wu X., Song M., Rakariyatham K. (2015). Anti-inflammatory effects of 4′-demethylnobiletin, a major metabolite of nobiletin. *Journal of Functional Foods*.

[84] Wu X., Song M., Rakariyatham K. (2015). Inhibitory effects of 4′-demethylnobiletin, a metabolite of nobiletin, on 12-*O*-tetradecanoylphorbol-13-acetate (TPA)-induced inflammation in mouse ears. *Journal of Agricultural and Food Chemistry*.

[85] Guo S., Qiu P., Xu G. (2012). Synergistic anti-inflammatory effects of nobiletin and sulforaphane in lipopolysaccharide-stimulated RAW 264.7 cells. *Journal of Agricultural and Food Chemistry*.

[86] Jang S.-E., Ryu K.-R., Park S.-H. (2013). Nobiletin and tangeretin ameliorate scratching behavior in mice by inhibiting the action of histamine and the activation of NF-*κ*B, AP-1 and p38. *International Immunopharmacology*.

[87] Nemoto K., Ikeda A., Yoshida C. (2013). Characteristics of nobiletin-mediated alteration of gene expression in cultured cell lines. *Biochemical and Biophysical Research Communications*.

[88] Lam K. H., Alex D., Lam I. K., Tsui S. K. W., Yang Z. F., Lee S. M. Y. (2011). Nobiletin, a polymethoxylated flavonoid from citrus, shows anti-angiogenic activity in a zebrafish in vivo model and HUVEC in vitro model. *Journal of Cellular Biochemistry*.

[89] Ma W., Feng S., Yao X., Yuan Z., Liu L., Xie Y. (2015). Nobiletin enhances the efficacy of chemotherapeutic agents in ABCB1 overexpression cancer cells. *Scientific Reports*.

[90] Fleisher B., Unum J., Shao J., An G. (2015). Ingredients in fruit juices interact with dasatinib through inhibition of BCRP: a new mechanism of beverage-drug interaction. *Journal of Pharmaceutical Sciences*.

[91] Kim H. J., Yonezawa T., Teruya T., Woo J.-T., Cha B.-Y. (2015). Nobiletin, a polymethoxy flavonoid, reduced endothelin-1 plus SCF-induced pigmentation in human melanocytes. *Photochemistry and Photobiology*.

[92] Kim J.-J., Korm S., Kim W.-S. (2014). Nobiletin suppresses MMP-9 expression through modulation of p38 MAPK activity in human dermal fibrobalsts. *Biological & Pharmaceutical Bulletin*.

[93] Roza J. M., Xian-Liu Z., Guthrie N. (2007). Effect of citrus flavonoids and tocotrienols on serum cholesterol levels in hypercholesterolemic subjects. *Alternative Therapies in Health and Medicine*.

[94] Aptekmann N. P., Cesar T. B. (2013). Long-term orange juice consumption is associated with low LDL-cholesterol and apolipoprotein B in normal and moderately hypercholesterolemic subjects. *Lipids in Health and Disease*.

[95] Seki T., Kamiya T., Furukawa K. (2013). Nobiletin-rich *Citrus reticulata* peels, a kampo medicine for Alzheimer's disease: a case series. *Geriatrics & Gerontology International*.

